# Human Anaplasmosis and *Anaplasma ovis* Variant

**DOI:** 10.3201/eid1606.090175

**Published:** 2010-06

**Authors:** Dimosthenis Chochlakis, Ioannis Ioannou, Yannis Tselentis, Anna Psaroulaki

**Affiliations:** University of Crete, Heraklion, Greece (D. Chochlakis, Y. Tselentis, A. Psaroulaki); Veterinary Services, Nicosia, Cyprus (I. Ioannou)

**Keywords:** Anaplasma ovis, anaplasmosis, bacteria, rickettsia, ticks, zoonoses, vector-borne infections, humans, Cyprus, letter

**To the Editor:** Anaplasmosis is a disease caused by bacteria of the genus *Anaplasma*. *A*. *marginale*, *A*. *centrale*, *A*. *phagocytophilum*, *A*. *ovis*, *A*. *bovis*, and *A*. *platys* are obligate intracellular bacteria that infect vertebrate and invertebrate host cells. *A. ovis*, which is transmitted primarily by *Rhipicephalus bursa* ticks, is an intraerythrocytic rickettsial pathogen of sheep, goats, and wild ruminants (*1*).

*Anaplasma* spp. infections in humans have been reported in Cyprus (*2*,*3*). We report infection of a human with a strain of *Anaplasma* sp. other than *A. phagocytophilum*, which was detected by PCR amplification of anaplasmatic 16S rRNA, major surface protein 4 (*msp4*), and heat shock protein 60 (*gro*EL) genes.

A 27-year-old woman was admitted to the pathology clinic of a hospital in Famagusta, Cyprus on May 14, 2007, with an 11-day history of fever (<39.5°C) after a tick bite. Before admission, the patient was treated with cefixime (400 mg/d for 3 days) and cefradine (2 g/d for 2 days) without abatement of the fever. Physical examination showed hepatosplenomegaly and an enlarged lymph node.

Initial laboratory examinations showed moderate anemia (hemoglobin 11.5 g/dL), thrombocytopenia (95,000 thrombocytes/mm^3^), increased levels of transaminases (aspartate aminotransferase 178 U/L, alanine aminotransferase 313 U/L, γ-glutamyl transferase 79 U/L, lactate dehydrogenase 698 U/L), an increased level of C-reactive protein (10.4 mg/L), and an increased erythrocyte sedimentation rate (80 mm/h). Blood samples were obtained from the patient at the time of admission and 7 days and 3 months later. Results of blood and urine cultures were negative for bacteria. A chest radiograph, computed tomography of the abdomen, and an echocardiograph of the heart showed unremarkable results. Blood samples were negative for antibodies against cytomegalovirus, Epstein-Barr virus, hepatitis, HIV, mycoplasma, coxackie virus, adenovirus, parvovirus, *Coxiella burnetii*, *R*. *conorii*, and *R*. *typhi*, and for rheumatoid factors. A lymph node biopsy specimen was negative for infiltration and malignancy. After treatment with doxycycline (200 mg/day for 11 days), ceftriaxone (2 g/day for 5 days), and imipenem/cilastatin (1,500 mg/day for 1 day), the patient recovered and was discharged 17 days after hospitalization.

Three serum samples from the patient were tested in Crete, Greece, for immunoglobulin (Ig) G and IgM against *A. phagocytophilum* antigen by using an immunofluorescent antibody assay (Focus Diagnostics, Cypress, CA, USA). Serologic analysis showed IgG titers of 0, 0, and 128 and IgM titers of 20, 20, and 20 against *A. phagocytophilum* in the 3 serum samples, respectively.

Because the blood samples were transported frozen, detection of morulae was not possible. DNA was extracted by using the QIAamp DNA Blood Mini Kit (QIAGEN, Hilden, Germany). PCR amplifications (MyCycler DNA thermal cycler; Bio-Rad, Hercules, CA, USA) were conducted for the anaplasmatic 16S rRNA gene; *A*. *marginale*, *A*. *centrale*, and *A*. *ovis* heat shock protein 60 (*groEL)* genes; and *A*. *marginale*, *A*. *centrale*, and *A*. *ovis* major surface protein 4 (*msp4*) genes (*4*,*5*). DNA from previous studies in Cyprus (*4*,*5*) was used as a positive control. Double-distilled water was used as a negative control.

PCR amplicons were purified by using the QIAquick Spin PCR Product Purification Kit (QIAGEN) and sequenced on a 4200 double-beam automated sequencer (LI-Cor, Inc., Lincoln, NE, USA). Sequences were processed by using ClustalW2 software (www.ebi.ac.uk/Tools/clustalw2/index.html) and the GenBank/European Molecular Biology database library (http://blast.ncbi.nlm.nih.gov/Blast.cgi). Phylogenetic trees were constructed by using MEGA 4 software (www.megasoftware.net).

The first blood sample was positive for *A*. *ovis* by PCR; the other 2 were negative. A 16S rRNA gene sequence (EU448141) from the positive sample showed 100% similarity with other *Anaplasma* spp. sequences (*A*. *marginale*, *A*. *centrale*, *A. ovis*) in GenBank. *Anaplasma* sp. *groEL* and *msp4* genes showed a 1,650-bp sequence (FJ477840, corresponding to 748 of 1,650 bp) and an 852-bp sequence (FJ460443) for these genes, respectively. Phylogenetic trees (Figure) were constructed by using *A*. *ovis* strains detected in sheep and goats in Cyprus (*5*).

**Figure:**
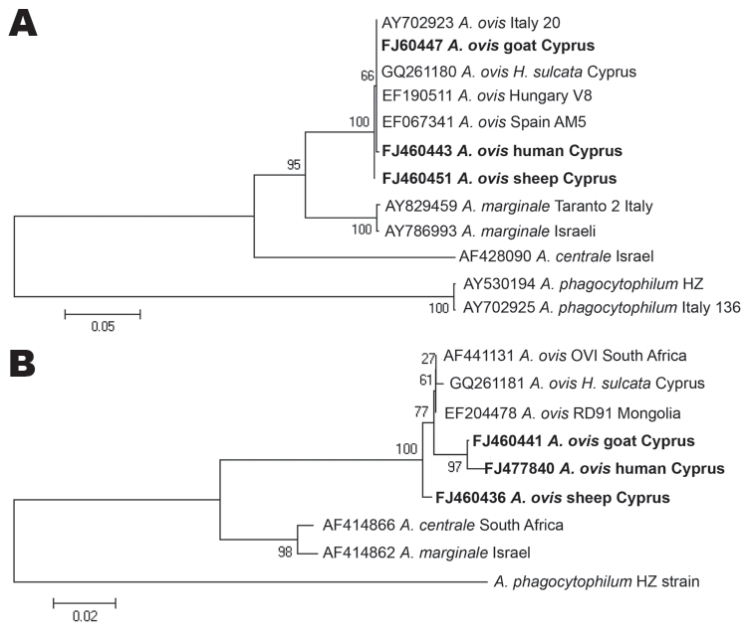
Evolutionary trees based on major surface protein 4 (A) and heat shock protein 60 (B) genes sequences of *Anaplasma phagocytophilum*, *A. marginale*, and *A. ovis*. Evolutionary history was inferred by using the neighbor-joining method. *H*. *sulcata*; *Haemaphysalis sulcata*. A) Optimal tree (branch length = 0.87919908) is shown. Percentages of replicate trees in which the associated taxa clustered together in the bootstrap test (500 replicates) are shown. B) Optimal tree (branch length = 0.34047351) is shown. Percentages of replicate trees in which the associated taxa clustered together in the bootstrap test (1,000 replicates) are shown. Trees are drawn to scale, with branch lengths in the same units as those of the evolutionary distances used to infer the phylogenetic tree. Evolutionary distances were computed by using the Kimura 2-parameter method. Strains detected in Cyprus are indicated in **boldface**. Scale bars indicate number of base substitutions per site.

Fever is common in cases of human infection with *A*. *phagocytophilum* (*6*). We also detected thrombocytopenia and elevated levels of transaminases. However, hepatosplenomegaly, lymphadenopathy, and anemia are not common in persons infected with *A*. *phagocytophilum*.

Immunofluorescent antibody analysis showed weak antibody titers against *A*. *phagocytophilum*. Serologic cross-reactivity of *Anaplasma* spp. is caused by conservation of major surface protein sequences (*7*).

*A*. *phagocytophilum* infection usually resolves after treatment with doxycyline for 4 days (*8*). The patient reported here was treated with doxycycline for 11 days. However, 1 case is not sufficient to form conclusions on severity and duration of illness.

In a study conducted in Cyprus, *Anaplasma* sp. was identified in birds (*9*). Because birds may be carriers of zoonotic pathogens, infection of humans with these pathogens may occur. However, transmission of *A*. *ovis* to humans is unclear. The role of *R. bursa*, a common tick species in sheep and goats in Cyprus (D. Chochlakis, unpub. data), as a vector of other pathogens for humans has been proposed (*10*). Whether these pathogens include *A*. *ovis* is unknown. Thus, laboratory testing of human blood samples should include universal primers against all *Anaplasma* spp. to avoid missing cases such as the one we report.
